# Novel loci and Mapuche genetic ancestry are associated with pubertal growth traits in Chilean boys

**DOI:** 10.1007/s00439-021-02290-3

**Published:** 2021-05-28

**Authors:** Lucas Vicuña, Tomás Norambuena, José Patricio Miranda, Ana Pereira, Veronica Mericq, Linda Ongaro, Francesco Montinaro, José L. Santos, Susana Eyheramendy

**Affiliations:** 1 Faculty of Engineering and Sciences, Universidad Adolfo Ibáñez, Peñalolén, Santiago, Chile; 2 Instituto Milenio de Investigación Sobre los Fundamentos de los Datos (IMFD), Santiago, Chile; 3 Institute of Nutrition and Food Technology, University of Chile, Santiago, Chile; 4 Institute of Maternal and Child Research, Faculty of Medicine, University of Chile, Santiago, Chile; 5 Estonian Biocentre, Institute of Genomics, University of Tartu, Riia 23b, 51010 Tartu, Estonia; 6 Department of Nutrition, Diabetes and Metabolism, School of Medicine, Pontificia Universidad Católica de Chile, Santiago, Chile

## Abstract

Puberty is a complex developmental process that varies considerably among individuals and populations. Genetic factors explain a large proportion of the variability of several pubertal traits. Recent genome-wide association studies (GWAS) have identified hundreds of variants involved in traits that result from body growth, like adult height. However, they do not capture many genetic loci involved in growth changes over distinct growth phases. Further, such GWAS have been mostly performed in Europeans, but it is unknown how these findings relate to other continental populations. In this study, we analyzed the genetic basis of three pubertal traits; namely, peak height velocity (PV), age at PV (APV) and height at APV (HAPV). We analyzed a cohort of 904 admixed Chilean children and adolescents with European and Mapuche Native American ancestries. Height was measured on roughly a $6-$month basis from childhood to adolescence between 2006 and 2019. We predict that, in average, HAPV is 4.3 cm higher in European than in Mapuche adolescents (*P* = 0.042), and APV is 0.73 years later in European compared with Mapuche adolescents (*P* = 0.023). Further, by performing a GWAS on 774, 433 single-nucleotide polymorphisms, we identified a genetic signal harboring 3 linked variants significantly associated with PV in boys (*P* $< 5 \times 10^{-8}$). This signal has never been associated with growth-related traits.

**Supplementary Information:**

The online version supplementary material available at 10.1007/s00439-021-02290-3.

## Introduction

Puberty is a complex developmental process characterized by distinct as well as shared phenotypes in males and females. For instance, while in boys voice breaking represents a developmental milestone, in females distinct pubertal markers are menarche, namely the onset of the first menstrual bleed (Day et al. 2015a); and the onset of breast development. Among shared pubertal phenotypes in males and females are the onset of pubic hair as well as the pubertal growth spurt, also called peak height velocity (PV), which is the period where maximum rate of growth occurs (Biro et al. 2006). Importantly, deviations from normal pubertal growth correlate with adult risk for certain kinds of cancer, diabetes and cardiometabolic disorders (Cousminer et al. 2013; Day et al. 2015b).

Pubertal parameters are also affected by genetic ancestry. For example, the age at onset of puberty, defined as the age at specific areolar and pubic hair stages, is earlier in African American girls than in European American girls (9.6 vs. 10.2 years, respectively). This is also true for the age at PV (APV; 11.5 vs. 11.9 years), age at menarche (12.0 vs. 12.6 years) and the age at attainment of adult height (16.5 vs. 17.1 years) (Biro et al. 2006). Maternal obesity significantly affects the onset of pubic hair in Asian and non-Hispanic girls, but not in African American girls. Among boys, the onset of pubic hair development occurs earlier in African Americans than in European Americans (Euling et al. 2008). However, such studies present some drawbacks. (i) Ancestry is considered as that reported by the participants’ parents and is defined as a categorical variable, thus not accounting for different degrees of genetic admixture. (ii) Social and cultural disparities that correlate with phenotypic differences between ethnic groups are usually not rigorously accounted for (Idossa et al. 2018). (iii) Most studies have analyzed populations with African and European ancestries. Hence, it is not known whether or not—and to which extent—the observed results hold true for other continental groups, such as populations with Native American ancestry.

The onset of puberty is triggered by a combination of genetic and environmental factors. Environmental factors account for $\sim 20-30\%$ of the variance of puberty’s onset and include nutrition, stress, life setting (urban vs. rural) and social status (Soliman et al. 2014; Herbison 2007; Parent et al. 2003). However, the major determinants of pubertal growth variability are genetic, since its heritability is 60–90% (Cousminer et al. 2013).

Genome-wide association studies (GWAS) have been key in identifying genetic variation involved in traits that result from body growth, such as adult height, which is highly heritable and polygenic, involving $>3,000$ genetic variants (Chan et al. 2015; Styrkarsdottir et al. 2019). However, it is mostly unknown how these variants influence height growth across distinct growth phases and populations (Paternoster et al. 2011; Mills and Rahal 2020). A few GWA studies performed on European populations have identified loci involved in height growth traits. For example, a GWA meta-analysis performed on a pooled set of European cohorts identified loci involved in the take-off phase of the growth spurt (height at 10 years in girls and 12 years in boys), the total magnitude of height growth during the pubertal growth spurt, the timing of puberty and age at menarche (Elks et al. 2010; Cousminer et al. 2013). Nevertheless, with the exception of age at menarche (Cousminer et al. 2016), non-European populations have been poorly analyzed in such studies, making difficult to estimate the genetic contribution of associated loci in populations with other continental ancestries.

The Chilean population consists of admixed individuals whose continental ancestry comes from Europe ($52\%$ on average), Native America ($45\%$ on average) and Africa ($3\%$ on average) (Eyheramendy et al. 2015). Their Native American ancestry component is made up of two sub-components, mainly Mapuche (from the lowlands of Central-Southern Chile) and Aymara (from the Andes highlands of Northern Chile) to a lower extent (Vicuña et al. 2020). While the general aim of this study was to identify genetic factors involved in pubertal height growth, our specific aims were twofold: (i) identifying novel genetic loci associated with PV, APV and HAPV; (ii) estimating the contribution of genetic ancestry in the variability of these traits. We implemented a GWAS in an Chilean cohort of 904 admixed individuals with European and Mapuche Native American ancestry, for whom these traits were measured from childhood to adolescence on a 6-month basis between 2010 and 2019.

## Results

### Characterization of PV, APV and HAPV in Chilean children

**Fig. 1:**
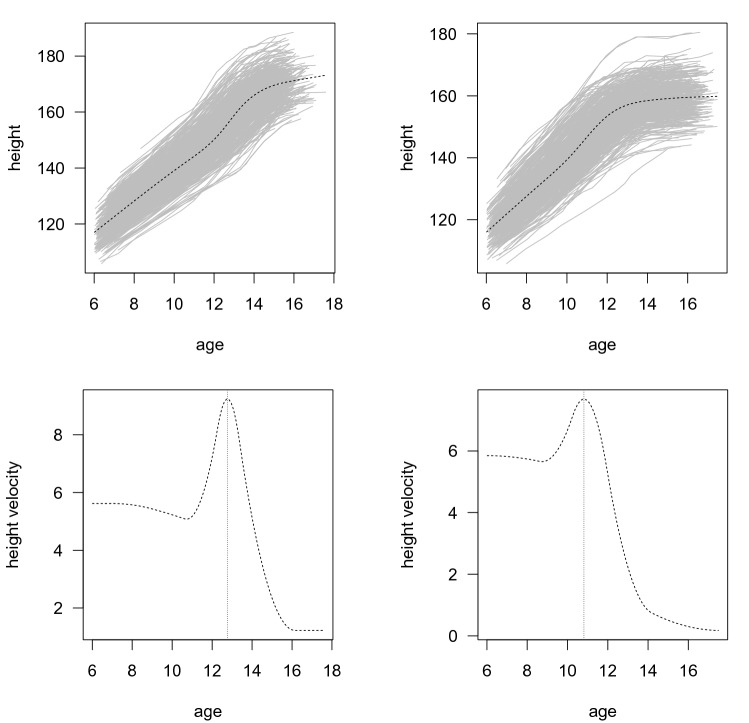
Estimation of PV and APV. The upper row shows the adjustment of the growth curves in boys (left) and girls (right), obtained using the SITAR package from R (Cao et al. 2018). The lower row shows the mean height velocity (cm/year) in boys (left) and girls (right). The dotted vertical lines pinpoint the APV

We modeled pubertal height growth in 440 Chilean boys and 464 girls separately. Figure 1 shows the growth curves for boys and girls (left and right panels in the top row, respectively) as well as the mean height velocity (left and right panels in the lower row, respectively). From the models, we estimated that the mean PV is 9.1 (±0.9 SD) cm/year in boys and 7.7 (±0.8 SD) cm/year in girls. The mean APV is 12.7 (±0.8 SD) years in boys and 10.8 (±0.8 SD) years in girls. We also found that HAPV is 156.4 (±5.5 SD) cm in boys and 145.1 (±5.4 SD) cm in girls (upper row).

### Global ancestry characterization of Chilean children

Because genetic ancestry can affect pubertal growth phenotypes, it is important to account for individual European and/or Native American ancestries in the GWAS for PV, APV and HAPV (next section). Indeed, not accounting for Mapuche and Aymara sub-ancestries has shown to markedly underestimate associations with disease phenotypes, as compared to when these sub-ancestries are combined together (Lorenzo Bermejo et al. 2017).

**Fig. 2:**
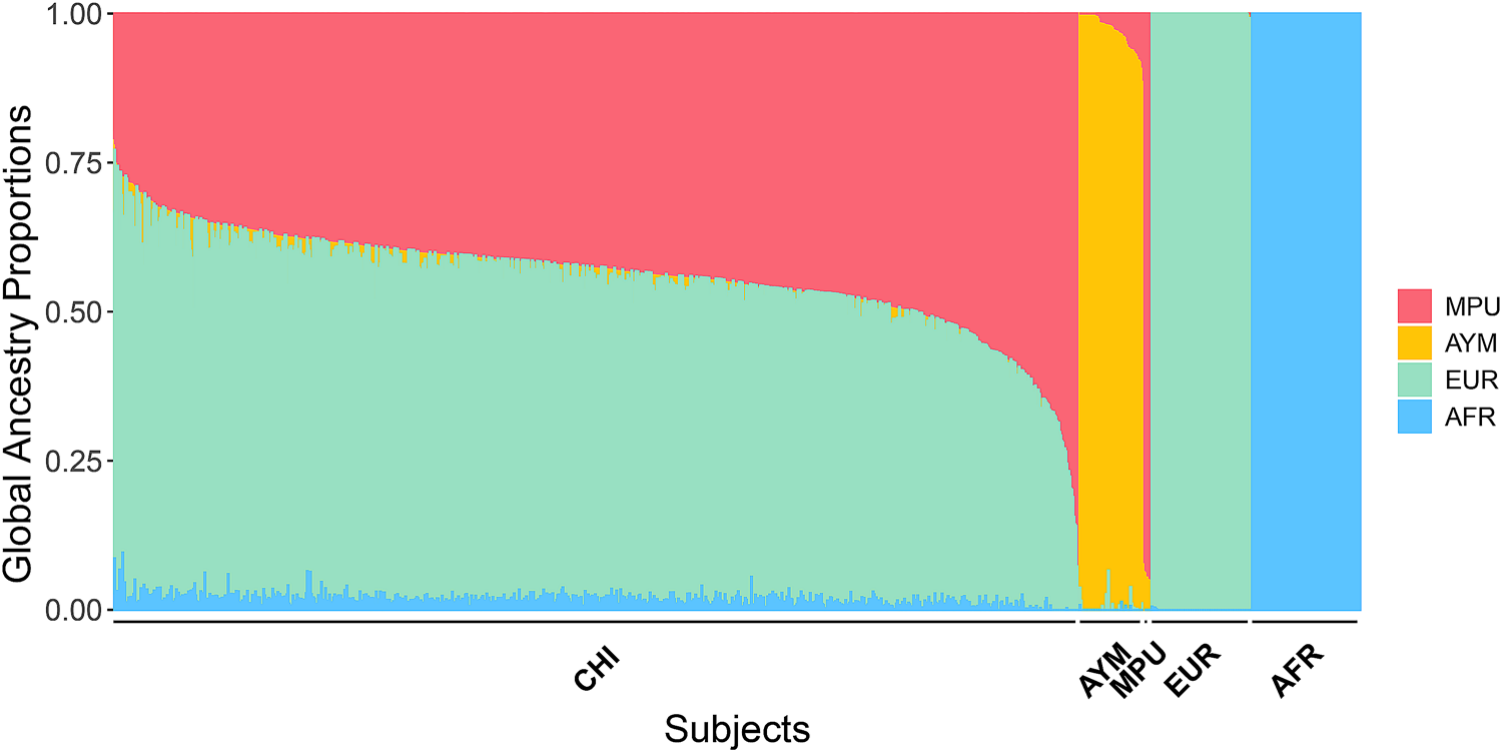
Global ancestry proportions. Each vertical line represents a subject and the colors the different ancestries. CHI: Chilean. The reference populations are MPU: Mapuche, AYM: Aymara, EUR: European; AFR: African

To our knowledge, neither comparisons in PV, APV and HAPV between European and Native American children, nor comparisons in pubertal growth between Mapuche and Aymara children have been formally tested. Therefore, we decided to account for the effect of European ancestry as well as Native-American sub-ancestries in the GWAS linear regressions for PV, APV and HAPV. Hence, we performed a global ancestry inference with ADMIXTURE (Alexander and Lange 2011), using $K=4$ ancestral populations. We chose this* K* value because it was able to clearly distinguish the Mapuche and Aymara subcomponents. As reference Native American populations, we used Mapuche and Aymara; as European and African reference populations we used CEU and YRI from 1000G, respectively (see Methods for details). Figure 2 shows the global ancestry proportions of each subject in our study, where each vertical line represents a child, and the colors the different ancestries. Individuals of the reference populations are also included. We found that the mean Native American ancestry proportion of our children is predominantly Mapuche (0.438), while their mean Aymara ancestry proportion is small (0.026). Also, our sample has 0.521 European and 0.015 African mean ancestry proportions, which is consistent with previous studies (Eyheramendy et al. 2015; Vicuña et al. 2020). Of note, admixture is homogeneous among individuals, with very few subjects having >80–90% of a single ancestry.

**Fig. 3:**
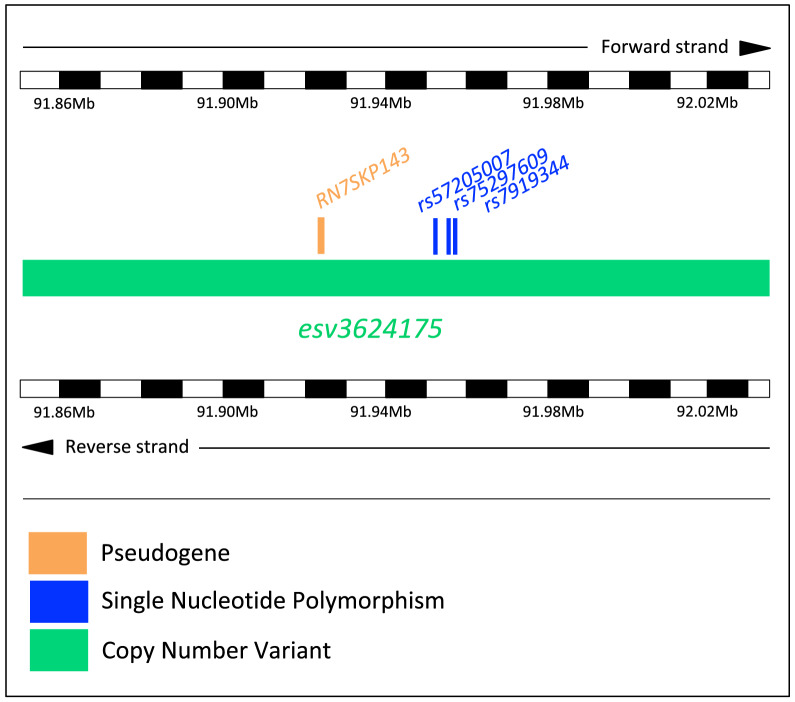
Genomic context of the genetic signal associated with PV in boys. Shown is the copy number variant *esv3624175* harboring the significantly associated variants *rs75297609*, *rs57205007* and *rs7919344* as well as the pseudogene *RN7SKP143*. The image includes chromosome bands intercalated between black and white blocks with 10 Kb of size

### GWAS of PV, APV and HAPV

We performed GWAS for PV, APV and HAPV. In the regression model, we adjusted for gender, Native American local ancestry, Mapuche global ancestry, genotype, an interaction effect between gender and genotype and an interaction effect between gender and Native American local ancestry. To increase the power to detect significant associations, all regressions were performed in boys and girls pooled together. Importantly, however, the interaction effects in our analyses allowed to separate the effects of the genotype and local ancestry on just boys, just girls or both (see Methods). In the analysis in boys, we found 2 variant alleles, *rs75297609-T* and *rs57205007-G*, achieving the genome-wide association significance threshold of *P* < 5 x 10$^{-8}$ for PV (Table 1). These two variants are located close to the *RN7SKP143* gene (Table 1), a pseudogene of the *7SK* gene with unknown function. The 2 associated variants, as well as *RN7SKP143*, are in turn located within the copy number variant (CNV) *esv3624175* (Fig. 3), and thus likely represent the same association signal. Indeed, the genotypes of *rs75297609* and *rs57205007* among individuals show a Pearson’s correlation coefficient of 0.78. *rs75297609-T* and *rs57205007-G* have 8 and 13 carriers, respectively, as well as allele frequencies of 0.004 and 0.007, respectively. Table 1 also shows 6 variants achieving nominal significance (*P* <10$^{-6}$).

**Fig. 4:**
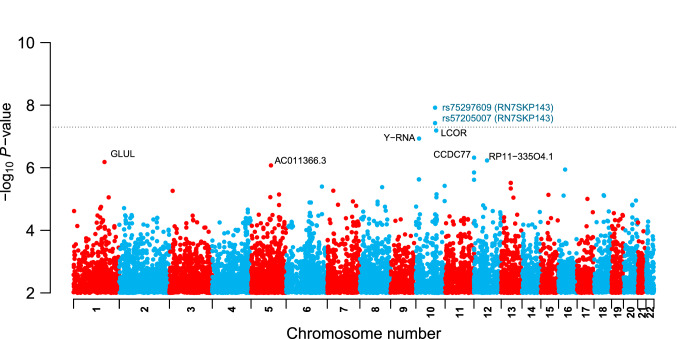
Manhattan plot for PV in boys. Genome-wide association *P* values, expressed as − log$_{10}$(*P* value). Highlighted in blue are the 2 variants achieving genome-wide significant associations (with the associated gene names within parentheses) as well as the genes most closely located to variants with *P*-value <10$^{-6}$ (in black). Variants with − log$_{10}$(*P*-value) between 0 and 2 are not shown

We did not find significant associations for APV. However, one variant, *rs148840332*, located in the *RP11-184E9.1* lincRNA gene, was associated with APV at *P* $=$3 x 10$^{-7}$ (Supplementary Table 1). In the GWAS for HAPV we did not find associations achieving nominal significance (*P* <10$^{-6}$; data not shown). In the analysis in girls (see Methods), we did not find significant associations for any phenotype. The Manhattan plots for the PV and APV GWAS are shown in Fig. 4 and Supplementary Fig. 1, respectively.

**Table 1:** Strongest associations for PV in boys

SNP ID-Allele	Conseq	Biotype	Gene	$\upbeta _{\mathrm{GT}}$	$P_{\mathrm{GT}}$	$\upbeta _{\mathrm{GTxSex}}$	$P_{\mathrm{GTxSex}}$	$\upbeta _{\mathrm{GTxLA}}$	$P_{\mathrm{GTxLA}}$
*rs75297609-T*	Upstream	miscRNA	*RN7SKP143* (98Kb)	− 2.72	1.2E-08	3.17	2.3E-06	2.08	5.4E-03
*rs57205007-G*	Upstream	miscRNA	*RN7SKP143* (86Kb)	− 2.13	3.7E-08	2.27	2.2E-06	1.13	2.4E-03
*rs113497890-G*	Intron	Protein	*LCOR*	− 3.16	6.3E-08	3.05	1.9E-05	− 0.45	6.2E-01
*rs12240935-C*	Upstream	miscRNA	$YRNA$ (24Kb)	− 3.09	1.2E-07	2.91	3.6E-03	2.36	3.9E-03
*rs143615510-A*	Intron	Protein	*CCDC77*	3.60	4.7E-07	− 2.66	1.2E-03	− 1.86	2.3E-02
*rs79659566-A*	Upstream	Protein	*DYRK2* (37Kb)	− 0.92	5.5E-07	0.41	2.4E-02	0.51	2.0E-05
*rs6699943-C*	Intron	Protein	*GLUL*	1.89	6.6E-07	− 1.15	1.4E-02	− 2.35	3.0E-07
*rs112103074-A*	Intergenic	–	*AC011366.3* (− 197 Kb)	− 1.21	8.3E-07	1.47	3.6E-06	0.76	1.7E-02

Shown are the SNP rs ID with the associated allele, the sequence ontology consequence type, biotype, closest gene (with the distance from the gene in Kb), effect size of the genotype ($\beta _{GT}$), association *P*-value of the genotype (*P*$_{GT}$), effect size of the interaction between genotype and sex ($\beta _{GTxSex}$) with the corresponding association *P*-value (*P*$_{GTxSex}$), effect size of the interaction between genotype and Native American local ancestry ($\beta _{GTxLA}$) with the corresponding association *P*-value (*P*$_{GTxLA}$). The coding for sex was 1 for females and 0 for males. Therefore, the effect of the SNP allele in males corresponds to the $\beta _{GT}$ column, and the effect of the SNP allele in females is the sum of the effects in the $\beta _{GT}$ and $\beta _{GTxSex}$ columns. Note that these effects are opposite in sign, which leads to a non-significant effect for females. Local ancestry is coded as 0, 1 or 2 depending on the number of alleles at each SNP that originate from Native America

**Fig. 5:**
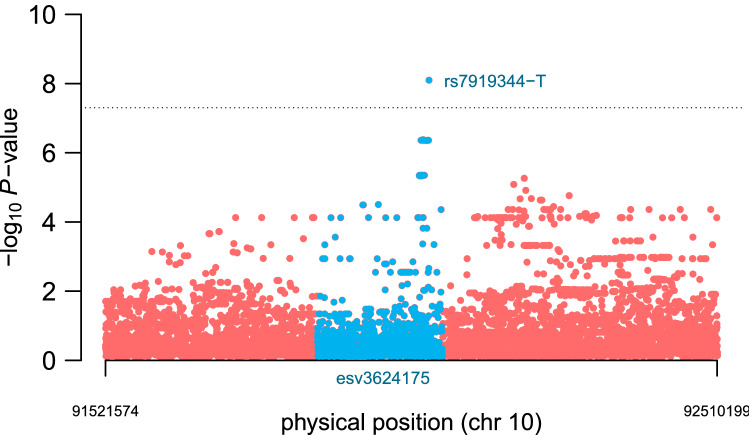
Manhattan plot for PV in imputed SNPs from the region surrounding the copy number variant *esv3624175*. The region shown encompasses $\pm 500$ Kb from *rs75297609* and *rs57205007*. Its physical coordinates are 10:91521574-92510199. Genome-wide association *P* values are expressed as − log$_{10}$(*P*-value). Highlighted in blue is *rs7919344-T*, the significantly associated variant allele located within *esv3624175*. All variants mapping *esv3624175* are labeled in blue. Variants with association *P* value $<10^{-5}$ (Table 2) also map *esv3624175* (not shown)

**Fig. 6:**
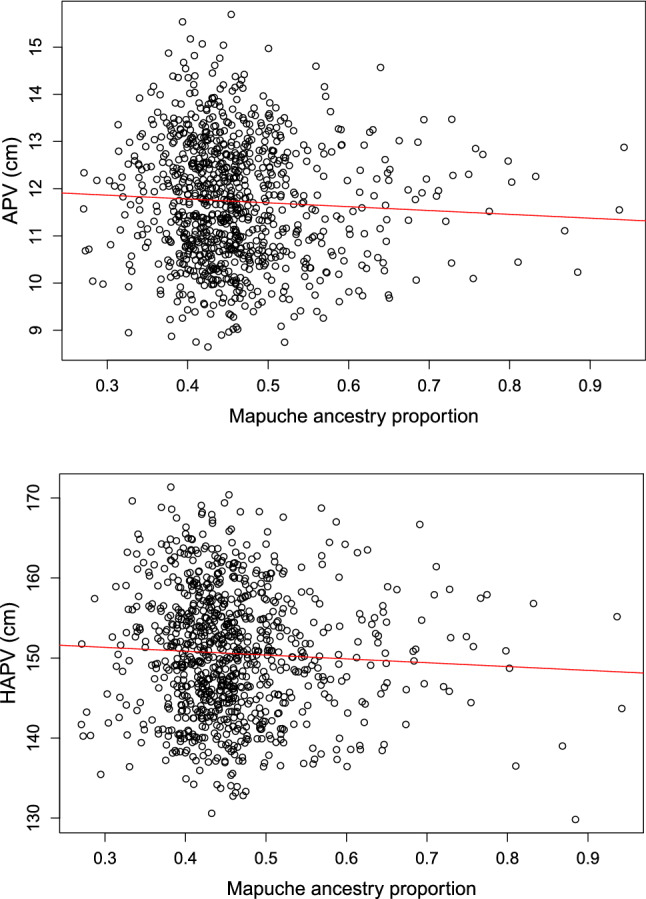
Effect of global Mapuche ancestry on APV and HAPV. The top and lower panels show how APV and HAPV vary as a function of global Mapuche ancestry proportions in boys and girls considered together. The regression line is labeled in red

To interrogate more variants for putative associations with PV, we performed genotype imputations of SNPs located within $\pm 500$ Kb of the association signal harboring *rs75297609* and *rs57205007*. We used the Trans-Omics for Precision Medicine (TOPMed) server (Taliun et al. 2021), which has a panel of 97, 256 samples from diverse ethnic backgrounds, including South Americans. We performed linear regressions adjusting for gender, Mapuche global ancestry, genotype and an interaction effect between gender and genotype. We did not include Native American local ancestry as covariate, since we lacked this information for imputed SNPs. In the analysis in boys, we found that the intergenic variant allele *rs7919344-T* was significantly associated with PV (*P* $= 1 \times 10^{-9}$; Fig. 5 and Table 2). We also found 7 intergenic SNPs achieving nominal genome-wide significance (*P* $< 10^{-6}$; Table 2), including the previously associated *rs75297609-T* variant allele. All of these variants seem to be part of the same peak (Fig. 5). Indeed, *rs7919344* is located 399 bp from *rs75297609* and 11.8 Kb from *rs57205007*. *rs7919344-T* has 7 carriers and an allele frequency of 0.004. In the analysis in girls, we did not find significant associations between PV and the imputed SNPs from chromosome 10.

**Table 2:** Stronger associations for PV in boys using imputed genotypes surrounding the association signal

SNP ID-Allele	Location	Gene	$\upbeta _{\mathrm{GT}}$	$P_{\mathrm{GT}}$	$\upbeta _{\mathrm{GTxSex}}$	$P_{\mathrm{GTxSex}}$
*rs7919344-T*	92021973	*RN7SKP143* (98 Kb)	− 2.38	8.0E-09	2.86	4.9E-06
*rs148396815-C*	92012923	*RN7SKP143* (89 Kb)	− 1.87	4.2E-07	2.60	6.1E-07
*10:92014488-T*	92014488	*RN7SKP143* (91 Kb)	− 1.87	4.4E-07	2.35	9.2E-05
*rs184996157-A*	92018837	*RN7SKP143* (95 Kb)	− 1.87	4.4E-07	2.35	9.2E-05
*rs534950014-G*	92011098	*RN7SKP143* (87 Kb)	− 1.87	4.4E-07	2.35	9.2E-05
*rs560385300-T*	92013183	*RN7SKP143* (89 Kb)	− 1.87	4.4E-07	2.35	9.2E-05
*rs75297609-T*	92021574	*RN7SKP143* (98 Kb)	− 1.87	4.4E-07	2.35	9.2E-05
*10:92016131-A*	92016131	*RN7SKP143* (92 Kb)	− 1.87	4.4E-07	2.08	1.7E-04

Shown is the SNP rs ID with the associated allele, physical location, gene, effect size of the genotype ($\beta _{GT}$), association *P* value of the genotype (*P*$_{GT}$), effect size of the interaction between genotype and sex ($\beta _{{GT}\times {Sex}}$) with the corresponding association *P* value (*P*$_{GT\times Sex}$). The effect of the SNP allele in males corresponds to the $\beta _{GT}$ column, and the effect of the SNP allele in females is the sum of the effects in the $\beta _{GT}$ and $\beta _{GT\times Sex}$ columns. Since these effects are opposite in sign, this leads to a non-significant effect for females. The region analyzed has physical coordinates 10:91521574-92510199

### Effect of genetic ancestry on pubertal growth phenotypes

We analyzed whether PV, APV and/or HAPV are influenced by global genetic ancestry proportions. We found that Mapuche Native American global ancestry had average effect sizes of – 0.73 years (*P* = 0.02) for APV and − 4.3 cm (*P* = 0.04) for HAPV in boys and girls combined (Fig. 6). Global ancestry did not have a significant effect on PV.

We also evaluated the effect of per-SNP wise local ancestry over PV and APV in non-imputed genotypes (HAPV was not analyzed due to an absence of strong GWAS associations). In the regression model, we measured the effect of Native American local ancestry and also the interaction term between genotype and Native American local ancestry (see Methods). The variant allele with the strongest genome-wide association with PV for the interaction term between genotype and Native American local ancestry was *rs6699943-C* (*P* $= 3 \times 10^{-7}$; Table 1). We also found strong associations for the two significant variants in the GWAS for PV (*P* = 0.002 for *rs57205007-G* and *P* = 0.005 for *rs75297609-T*; Table 1).

## Discussion

In this study, we aimed to better understand the genetic architecture of pubertal growth. We conducted GWAS on PV, APV and HAPV. The two former traits are critical antropometric markers of pubertal growth (Granados et al. 2015; Sovio et al. 2009). However, besides a few association studies between candidate genetic variants and PV in Europeans (Sovio et al. 2009), we are not aware of previous GWAS on PV, APV or HAPV.

Our GWAS on called and imputed SNPs detected a single genetic signal overlapping the copy number variant (CNV) *esv3624175*, which harbors the significantly associated variants *rs75297609-T*, *rs57205007-G* and *rs7919344-T*. This suggests that a causal variant linked to this region is driving the association with PV. This hypothesis is also supported by the high correlation across the genotypes of *rs75297609* and *rs57205007*. The associated variants are located close to the *RN7SKP143* pseudogene, which has unknown function. Even though the biological functions of the >200 *7SK* pseudogenes are unknown, some of them might have growth-related functions. For instance, on the same chromosome 10, *RN7SKP167* has been GWAS-associated with growth-related phenotypes, including bone mineral density and hip bone size (*P* = $4 \times 10^{-11}$) (Zhang et al. 2020). However, most pseudogenes are not functional, so it is also possible that the three significant GWAS associations could result from these variants being in high linkage disequilibrium with causal variants located in or around other growth-related genes. Unfortunately, there is no recombination map for the Chilean population to test this hypothesis.

We found an intron variant allele of the *LCOR* gene, namely, *rs113497890-G*—also on chromosome 10—reaching a *P* value very close to genome-wide significance (*P* $= 6.3 \times 10^{-8}$). This gene has been GWAS-associated with adult height (*P* $= 4 \times 10^{-20}$) and heel bone mineral density (*P* $= 1 \times 10^{-8}$) (Kichaev et al. 2019), two phenotypes likely related with pubertal height growth. Increasing our sample size may augment statistical power to detect a significant association of the *LCOR* variant. It remains to be elucidated whether the main effects of the variants associated with PV on body growth would result from their neuro-endocrine actions through the hypothalamic–pituitary–gonadal (HPG) axis that triggers puberty (Bordini and Rosenfield 2011), or rather over local growth factors responsible for tissue growth. It is also unclear what biological mechanism underlies the sex-specific effects of the associated variants.

We analyzed the relationship between longitudinal pubertal traits and genetic ancestry. There are very few studies addressing this topic. An epidemiological study performed in a multiethnic US cohort showed that the mean APV is 13.7 years in boys and 12.1 years in females (Granados et al. 2015). Our results show that Chilean children have a lower mean APV (12.7 years in boys and 10.8 years in girls). However, since that study on US children did not consider genetic variables, it is not possible to estimate whether or not ancestry contributes to these differences. In our study of Chilean children, whose range for Mapuche ancestry varied from 0.25 to 0.94, we obtained that a difference in ancestry proportion led on average to a 0.73 years decrease in APV between a European and a Mapuche Native American boy. To our knowledge, we are the first to quantify how Native American genetic ancestry affects pubertal growth phenotypes, specifically PV, APV and HAPV. It is also important to recognize that differences in height growth and puberty milestones by ethnicity are not only determined by genetic ancestry, but also are influenced by social disparities among ethnic groups (Idossa et al. 2018).

Mapuche and Aymara diverged from their common ancestral population $\sim$8750 years ago (Lindo et al. 2018). Thus, it is possible that long-time exposure to different environments as well as demographic forces led to changes in their genetic diversity, which in turn could have differentially affected pubertal growth-related phenotypes in a population-specific manner. However, to our knowledge, comparisons in pubertal growth between Mapuche and Aymara children have never been formally tested. By accounting for Mapuche and Aymara global ancestries in the GWAS linear regressions instead of considering them as a single ancestral component, we obtained more reliable GWAS associations, since most of the Native American ancestry of the Chilean children is Mapuche. Noteworthy, we found that the strongest genome-wide association for the interaction between genotype and local ancestry is *rs6699943-C*, an intron variant allele of the *GLUL* gene, which has not been GWAS associated with height growth-related traits.

Unfortunately, we did not replicate our findings in an independent cohort, since there is no other longitudinal paediatric growth cohort with admixed Native American and European ancestries. European cohorts such as those that comprise the Early Growth Genetics Consortium (Middeldorp et al. 2019), are not useful for replication, since the three associated variant alleles are only found in Africans and in Latino populations (when considering populations from 1000G) (The 1000 Genomes Project Consortium 2015). An exception is IBS (Spaniards) from 1000G, where *rs75297609-T* and *rs57205007-G* are found with allele frequencies of 0.005 and 0.009, respectively (information retrieved from the Variant Effect Predictor (McLaren et al. 2016). None of these variants are present in our Mapuche or Aymara reference panels. Future research will be needed to determine the ancestral origin of the genetic signal harboring the three associated variants.

The results of our study highlight the importance of including genetic ancestry when performing GWAS in populations of admixed ancestries, such as Latinos or African Americans. Finally, since pubertal growth traits significantly vary with genetic ancestry, GWAS on understudied longitudinal growth traits hold great potential to identifying loci playing functional roles in particular continental populations.

## Subjects and methods

### Sample

We analized individuals of the “Growth and Obesity Chilean Cohort Study” (GOCS) (Corvalán et al. 2013). This is a longitudinal follow-up of 1195 individuals recruited in 2006 (aged 3.5 years old) at different childcare centers from the South East Area of Santiago, Chile. The population represents a middle-low socioeconomic level and all participants were singleton births occurring during $2002-2003$, who had birth weights between 2500 and 4500 g, with no medical or mental conditions. Annual measurements were carried out at Instituto de Nutrición y Tecnología en Alimentos (INTA), Santiago, Chile, by trained dietitians, and since 2010 visits took place every 6 months. We used standardized protocols and height was measured with a portable stadiometer (Harpenden 603; Holtain LTD, Crosswell, UK) to the nearest 0.1 cm.

### Genotyping

We used genome-wide data of 848 individuals from Tobar et al. (2019) and genotyped 105 additional individuals for the present study. Genotype data was obtained using the Infinium $^{{\textregistered } }$ Multhi-Ethnic Global BeadChip (Illumina). Genotyping was performed in the Human Genotyping laboratory at the Spanish National Cancer Research Centre, a member of CeGen (PRB2-ISCIII). Raw intensity genotype files were loaded into GenomeStudio v2.0.3 (Illumina) and automatic clustering was performed. We removed 18 samples with call rate $<0.98$ from the total of samples genotyped. We also excluded variants with heterozygous genotypes on the X chromosome for males and variants calling genotypes on Y chromosome for females. Using PLINK 1.9 (Purcell et al. 2007) we removed 10 additional samples based on gender mismatch, heterozygosity rate (variants with ±3 SD from the mean heterozigosity) and ancestry outliers. For relatedness, we randomly discarded 1 person from each pair with IBD/IBS>0.2, corresponding to a kinship relationship between second and third degree. Excluded variants had missing genotype data $>5$%, duplicated physical positions (one variant was kept from each duplicate pair) and deviations from Hardy–Weinberg equilibrium (HWE) (*P* $< 1 \times 10^{-6}$). A-T and C-G tranversions were also excluded to avoid inconsistencies with the reference human genome (build GRCh37). We also excluded 25 boys whose last measurements were taken before they were 12 years old. After applying these filters, we obtained a clean data set of 904 individuals and 774, 433 autosomal variants.

### Local ancestry estimation

We used RFMix (Maples et al. 2013) to infer the local ancestry of genomic fragments in the 904 Chilean individuals. As reference populations for the local ancestry analysis we used the following populations from the 1000 Genomes Project (The 1000 Genomes Project Consortium 2015): Yoruba (YRI, $n = 108$) for African ancestry, Utah Residents with Northern and Western European Ancestry (CEU, $n = 99$) for European ancestry, and Peruvian (PEL, $n = 85$) for Native American ancestry. We merged all sets obtaining 1196 individuals and the 774, 433 SNPs. We inferred the gametic phase of individuals using Beagle 5 software (Browning et al. 2018), and the HapMap37 human genome build 37 recombination map. We used PopPhased -n 5 and –forward-backward parameters as recommended in the RFMix manual.

### Global ancestry estimation

We estimated the global ancestry proportions with ADMIXTURE (Alexander and Lange 2011), using K = 4 ancestral populations. As reference Native American populations, we included 7 Mapuche individuals from Vidal et al. (2019) and 64 Aymara individuals from Lindo et al. (2018) and Crawford et al. (2017). As proxies for the European and African components, we used 99 individuals with Northern/Western European ancestry (CEU) and 102 Yoruba individuals from the 1000G Project (The 1000 Genomes Project Consortium 2015), respectively.

### Genotype imputation

SNPs were imputed using the TOPMed Server (Taliun et al. 2021), which includes a panel of 97,256 deeply sequenced human genomes. Genotypes aligned to the GRCh37/h19 assembly were submitted into the server and imputations were run with default parameters. Imputed genotypes were converted from GRCh38 to GRCh37/h19 assembly using CrossMap (Zhao et al. 2014). Chain files as well as the reference genome were obtained from the UCSC Genome Browser (Kent et al. 2002). Imputed genotypes were filtered by a R-square value of 0.5. Indels were removed and assignment of Reference ID (RS number) was based on 1000G’s SNPs.

### Longitudinal model

We estimated the growth curves for each individual in the sample through non-linear longitudinal mixed models; one model fitted to the girls and another model fitted to the boys, as implemented in the R package SITAR (Cao et al. 2018). The model takes the form $y_{it}=\alpha _i+h(\frac{t-\beta _i}{exp(\gamma _i)})$, where $\alpha _i, \beta _i$ and $\gamma _i$ are random effects for individual *i*, *h* is a natural cubic spline function of age (*t* in the model) versus height, where $y_{it}$ represents the height of individual *i* ($i=1,\ldots ,n$) at time *t*. The fitted curves for each individual were used to calculate peak height velocity (PV), age at peak height velocity (APV) and height at the age of peak velocity (HAPV) using the function getapv (Cao et al. 2018).

### GWAS for PV, APV and HAPV

For each of the three phenotypes we performed genome-wide associations by implementing linear regression models. The model included boys and girls together. We adjusted for gender, Native American local ancestry at each SNP, global ancestry for each individual and the genotype for each SNP. Global ancestry refers to the proportion of global Mapuche Native American ancestry and local ancestry was measured for each child at each SNP, and it consists of an estimate of the number of alleles that originate from Native America. It takes values 0, 1 or 2. We also included in the model an interaction effect between genotype and local ancestry and an interaction effect between genotype and gender. The linear regression equation for the model was the following:

$$\begin{aligned} ph_i = \beta _0+ \beta _1X_{1i}+ \beta _2X_{2i}+ \beta _{3j}X_{3ij}+ \beta _{4j}X_{4ij}+\beta _{5j}X_{1i}\times X_{4ij}+\beta _{6j}X_{1i}\times X_{3ij}+\epsilon _i \end{aligned}$$

where $X_1$ is a dichotomous variable that represents male gender with 0 and female gender with 1. $X_2$ represents the proportion of global Mapuche Native American ancestry (this variable takes values between 0 and 1), $X_{3j}$ represents the local Native American ancestry at SNP *j* (takes values 0, 1 or 2 that represent the number of alleles that originate from Native Americans), and $X_{4j}$ corresponds to the additive genotype representation of SNP *j*. *ph* represents the phenotype that can take on the three phenotypes that we consider (PV, APV and HAPV) and the subscript *i* stands for measurements at individual *i*. Note that the genetic association of SNP *j* on female children is given by $\beta _{4j}+\beta _{5j}$, while the association on male children is $\beta _{4j}$. To identify significant genetic variants we performed three test of hypotheses for each phenotype and each SNP *j*: (i) $H_0: \beta _{4j}+\beta _{5j}=0 \text{ vs } H_1: \beta _{4j}+\beta _{5j}\ne 0$; (ii) $H_0: \beta _{4j}=0 \text{ vs } H_1: \beta _{4j}\ne 0$; (iii) $H_0: \beta _{5j}=0 \text{ vs } H_1: \beta _{5j}\ne 0$.

To test associations between imputed SNPs and PV, we implemented a regression model similar to the aforementioned model, but we excluded the effect of Native American local ancestry. The linear regression equation for the model was the following:

$$\begin{aligned} ph_i = \beta _0+ \beta _1X_{1i}+ \beta _2X_{2i}+ \beta _{4j}X_{4ij}+\beta _{5j}X_{1i}\times X_{4ij}+ \epsilon _i \end{aligned}$$

The symbols of the covariates, their effects and the hypotheses tested were described in the previous model.

### Variant and gene annotations

Variant annotations corresponding to the GRCh37 (hg19) assembly were retrieved with the web tool Variant Effect Predictor (VEP) from Ensembl (McLaren et al. 2016). They included the Sequence Ontology (SO) consequence type and Gencode biotypes. Upstream and downstream variants were defined as those located 10 Kb upstream or downstream of the gene, respectively. Intergenic variants were defined as those located >100 Kb upstream or downstream of the closest gene. We used HaploReg v4.1 (Ward and Kellis 2012) to identify the genes located closest to associated intergenic variants. Reported GWAS associations were retrieved from the NHGRI GWAS Catalog (Welter et al. 2014). Only variants achieving the genome-wide associations threshold of *P* $< 5 \times 10^{-8}$ were considered. When more than one variant in a gene has been associated with the same phenotype, we reported the strongest association.

## Supplementary Information

Below is the link to the electronic supplementary material.

Supplementary material 1 (pdf 176 KB)

Supplementary material 2 (pdf 623 KB)

## Data Availability

We can provide summary data upon request.
