# Supplementary material for: Novel loci and Mapuche genetic ancestry are associated with pubertal growth traits in Chilean boys
Source: Hum Genet. 2021 May 28;140(12):1651–61. doi: 10.1007/s00439-021-02290-3 (PMC8553699; doi:10.1007/s00439-021-02290-3)
Supplement: Supplementary file 1 — Supplementary material 1 (pdf 176 KB) [file 439_2021_2290_MOESM1_ESM.pdf]

SUPPLEMENTARY FILES

https://www.overleaf.com/project

Supplementary Figures

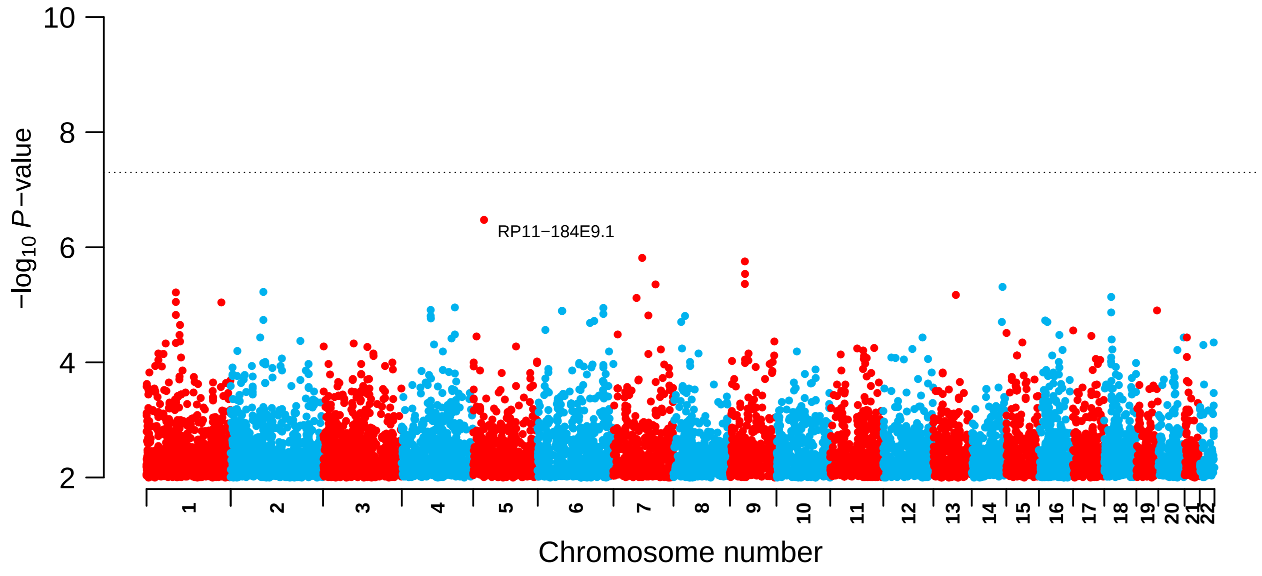

Supplementary Figure 1. Manhattan plot for APV in boys. Genome-wide association P-values, expressed as -$\log_{10}$(P-value). Highlighted are genes linked to variants with P-value $<10^{-6}$. Variants with -$\log_{10}(P\text{-value})$ between 0 and 2 are not shown.

Supplementary Tables

SNP ID-Allele	Conseq	Biotype	Gene	$ß_{GT}$	P_{GT}	$ß_{GTxSex}$	P_{GTxSex}	$ß_{GTxLA}$	P_{GTxLA}
rs148840332-C	intron/NCT	lincRNA	*RP11-184E9.1*	1.96	3.4E-07	-1.66	4.8E-04	-1.13	2.7E-02

Supplementary Table 1. Strongest association for APV in boys. Shown is the SNP rs ID with the associated allele, consequence type, biotype, gene, effect size of the genotype (β_{GT}), association P-value of the genotype (P_{GT}), effect size of the interaction between genotype and sex (β_{GTxSex}) with the corresponding association P-value (P_{GTxSex}), effect size of the interaction between genotype and Native American local ancestry (β_{GTxLA}) with the corresponding association P-value (P_{GTxLA}). The effect of the SNP allele in males corresponds to the β_{GT} column, and the effect of the SNP allele in females is the sum of the effects in the β_{GT} and β_{GTxSex} columns. Local ancestry is coded as 0, 1 or 2 depending on the number of alleles at each SNP that originate from Native America.
