# Supplementary material for: Novel loci and Mapuche genetic ancestry are associated with pubertal growth traits in Chilean boys
Source: Hum Genet. 2021 May 28;140(12):1651–61. doi: 10.1007/s00439-021-02290-3 (PMC8553699; doi:10.1007/s00439-021-02290-3)
Supplement: Supplementary file 2 — Supplementary material 2 (pdf 623 KB) [file 439_2021_2290_MOESM2_ESM.pdf]

Novel Loci and Mapuche Genetic Ancestry are Associated with Pubertal Growth Traits in Chilean Boys

Lucas Vicuña[1,2], Tomás Norambuena[1,2], José Patricio Miranda[1], Ana Pereira[3], Veronica Mericq[4], Linda Ongaro[5], Francesco Montinaro[5], José L. Santos [6] & Susana Eyheramendy[1,2,*]

[1]*Faculty of Engineering and Sciences, Universidad Adolfo Ibáñez, Peñalolén. Santiago, Chile.*

[2]*Instituto Milenio de Investigación Sobre los Fundamentos de los Datos (IMFD). Santiago, Chile.*

[3]*Institute of Nutrition and Food Technology, University of Chile. Santiago, Chile.*

[4]*Institute of Maternal and Child Research, Faculty of Medicine, University of Chile. Santiago, Chile.*

[5]*Estonian Biocentre, Institute of Genomics, University of Tartu, Riia 23b, 51010. Tartu, Estonia*

[6]*Department of Nutrition, Diabetes and Metabolism, School of Medicine, Pontificia Universidad Católica de Chile. Santiago, Chile.*

*Corresponding author: S.E. (email: susana.eyheramendy@uai.cl).

Keywords: GWAS, puberty, genetic ancestry, Native Americans

Puberty is a complex developmental process that varies considerably among individuals and populations. Genetic factors explain a large proportion of the variability of several pubertal traits. Recent genome-wide association studies (GWAS) have identified hundreds of variants involved in traits that result from body growth, like adult height. However, they do not capture many genetic loci involved in growth changes over distinct growth phases. Further, such GWAS have been mostly performed in Europeans, but we do not know how these findings relate to other continental populations. In this study, we analyzed the genetic basis of three pubertal traits; namely, peak height velocity (PV), age at PV (APV) and height at APV (HAPV). We analyzed a cohort of 904 admixed Chilean children and adolescents with European and Mapuche Native American ancestries. Height was measured on roughly a $6-$month basis from childhood to adolescence between 2006 and 2019. We predict that the difference in HAPV between an European and a Mapuche adolescent is 4.3 cm higher in the European (*P* = 0.042) and APV is 0.73 years later for the European compared with the Mapuche adolescent on average (*P* = 0.023). Further, by performing a GWAS on $774,433$ single nucleotide polymorphisms, we identified a genetic signal harboring 3 linked variants significantly associated with PV in boys ($P < 5 \times 10^{-8}$). This signal has never been associated with growth-related traits.

Introduction

Puberty is a complex developmental process characterized by distinct as well as shared phenotypes in males and females. For instance, while in boys voice breaking represents a developmental mile-

stone, in females distinct pubertal markers are menarche, namely the onset of the first menstrual bleed [1]; and the onset of breast development. Among shared pubertal phenotypes in males and females are the onset of pubic hair as well as the pubertal growth spurt, also called peak height velocity (PV), which is the period where maximum rate of growth occurs [2]. Importantly, deviations from normal pubertal growth correlate with adult risk for certain kinds of cancer, diabetes and cardiometabolic disorders [3] [4].

Pubertal parameters are also affected by genetic ancestry. For example, the age at onset of puberty, defined as the age at specific areolar and pubic hair stages, is earlier in African American girls than in European American girls (9.6 vs. 10.2 years, respectively). This is also true for the age at PV (APV; 11.5 vs. 11.9 years), age at menarche (12.0 vs. 12.6 years) and the age at attainment of adult height (16.5 vs. 17.1 years) [2]. Maternal obesity significantly affects the onset of pubic hair in Asian and non-Hispanic girls, but not in African American girls. Among boys, the onset of pubic hair development occurs earlier in African Americans than in European Americans [5]. However, such studies present some drawbacks. i) Ancestry is considered as that reported by the participants' parents and is defined as a categorical variable, thus not accounting for different degrees of genetic admixture. ii) Social and cultural disparities that correlate with phenotypic differences between ethnic groups are usually not rigorously accounted for [6]. iii) Most studies have analyzed populations with African and European ancestries. Hence, it is not known whether or not –and to which extent– the observed results hold true for other continental groups, such as populations with Native American ancestry.

The onset of puberty is triggered by a combination of genetic and environmental factors. Environmental factors account for $\sim 20 - 30\%$ of the variance of puberty's onset and include nutrition, stress, life setting (urban vs. rural) and social status [7–9]. However, the major determinants of pubertal growth variability are genetic, since its heritability is 60-90% [3].

Genome-wide association studies (GWAS) have been key in identifying genetic variation involved in traits that result from body growth, such as adult height, which is highly heritable and polygenic, involving $> 3,000$ genetic variants [10,11]. However, it is mostly unknown how these variants influence height growth across distinct growth phases and populations[12,13]. A few GWA studies performed on European populations have identified loci involved in height growth traits. For example, a GWA meta-analysis performed on a pooled set of European cohorts identified loci involved in the take-off phase of the growth spurt (height at 10 years in girls and 12 years in boys), the total magnitude of height growth during the pubertal growth spurt, the timing of puberty and age at menarche [14] [3]. Nevertheless, with the exception of age at menarche [15], non-European populations have been poorly analyzed in such studies, making difficult to estimate the genetic contribution of associated loci in populations with other continental ancestries.

The Chilean population consists of admixed individuals whose continental ancestry comes from Europe (52% on average), Native America (45% on average) and Africa (3% on average) [16]. Their Native American ancestry component is made from two sub-components, mainly Mapuche (from the lowlands of Central-Southern Chile) and Aymara (from the Andes highlands of Northern Chile) to a lower extent [17]. While the general aim of this study was to identify genetic factors

involved in pubertal height growth, our specific aims were twofold: i) Identifying novel genetic loci associated with PV, APV and HAPV; ii) Estimating the contribution of genetic ancestry in the variability of these traits. We implemented a GWAS in an Chilean cohort of 904 admixed individuals with European and Mapuche Native American ancestry, for whom these traits were measured from childhood to adolescence on a 6-months basis between 2010 and 2019.

Results

Characterization of PV, APV and HAPV in Chilean children We modeled pubertal height growth in 440 Chilean boys and 464 girls separately. **Figure** 1 shows the growth curves for boys and girls (left and right panels in the top row, respectively) as well as the mean height velocity (left and right panels in the lower row, respectively). From the models, we estimated that the mean PV is 9.1 ($\pm$0.9 SD) cm/year in boys and 7.7 ($\pm$0.8 SD) cm/year in girls. The mean APV is 12.7 ($\pm$0.8 SD) years in boys and 10.8 ($\pm$0.8 SD) years in girls. We also found that HAPV is 156.4 ($\pm$5.5 SD) cm in boys and 145.1 ($\pm$5.4 SD) cm in girls (**Figure** 1, upper row).

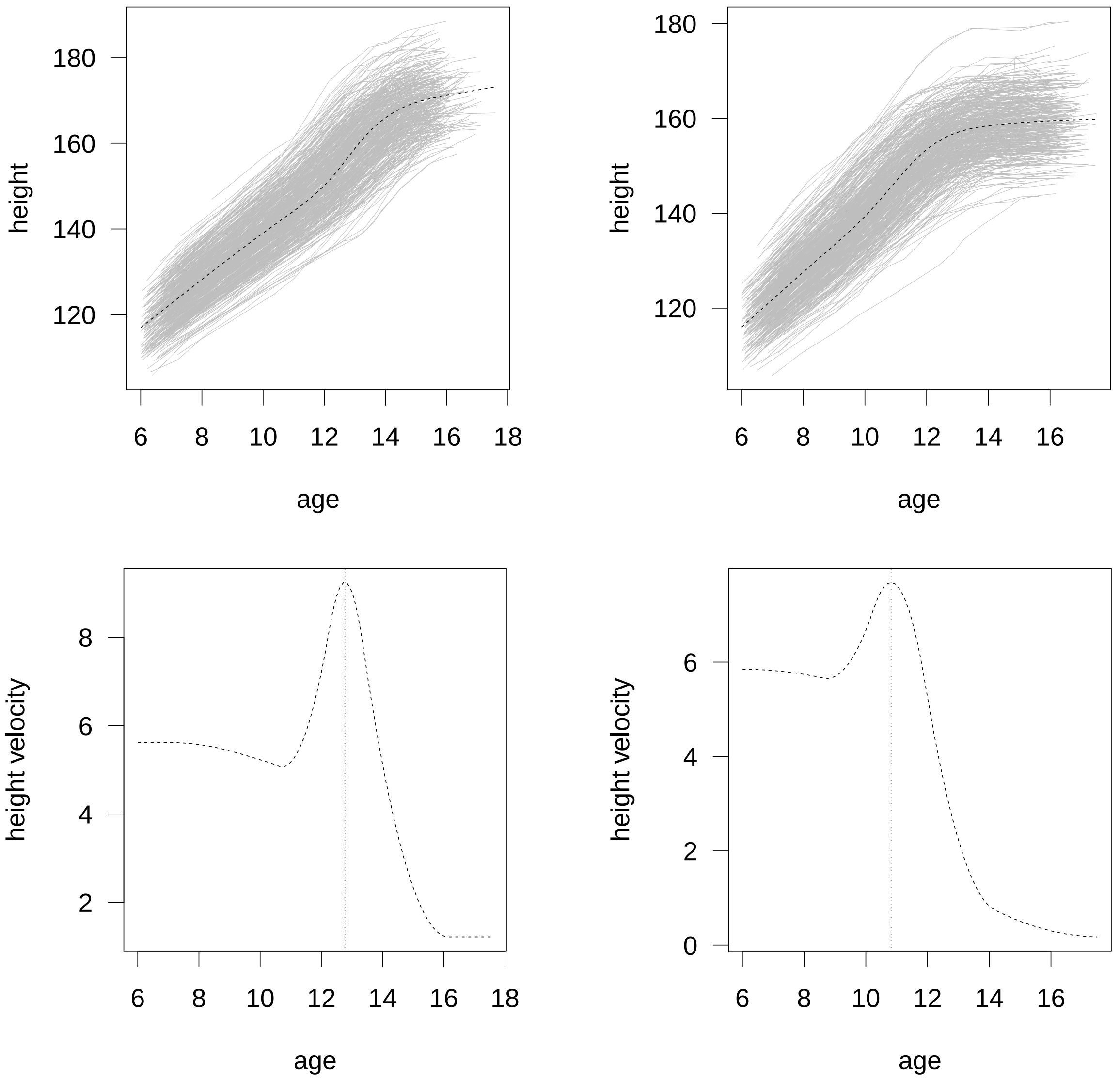

Global ancestry characterization of Chilean children. Because genetic ancestry can affect pubertal growth phenotypes, it is important to account for individual European and/or Native American ancestries in the GWAS for PV, APV and HAPV (next section). Indeed, not accounting for Mapuche and Aymara sub-ancestries has shown to markedly underestimate associations with disease phenotypes, as compared to when these sub-ancestries are combined together [18].

To our knowledge, neither comparisons in PV, APV and HAPV between European and Native American children, nor comparisons in pubertal growth between Mapuche and Aymara children have been formally tested. Therefore, we decided to account for the effect of European ancestry as well as Native-American sub-ancestries in the GWAS linear regressions for PV, APV and HAPV. Hence, we performed a global ancestry inference with ADMIXTURE [19], using K=4 ancestral populations. We chose this K value because it was able to clearly distinguish the Mapuche and Aymara subcomponents. As reference Native American populations we used Mapuche and Aymara; as European and African reference populations we used CEU and YRI from 1000G, respectively (see Methods for details). **Figure** 2 shows the global ancestry proportions of each subject in our study, where each vertical line represents a child, and the colors the different ancestries. Individuals of the reference populations are also included. We found that the mean Native American ancestry proportion of our children is predominantly Mapuche (0.438), while their mean Aymara ancestry proportion is small (0.026). Also, our sample has 0.521 European and 0.015 African mean ancestry proportions, which is consistent with previous studies [16] [17]. Of note, admixture is homogeneous among individuals, with very few subjects having $>$80-90% of a single ancestry.

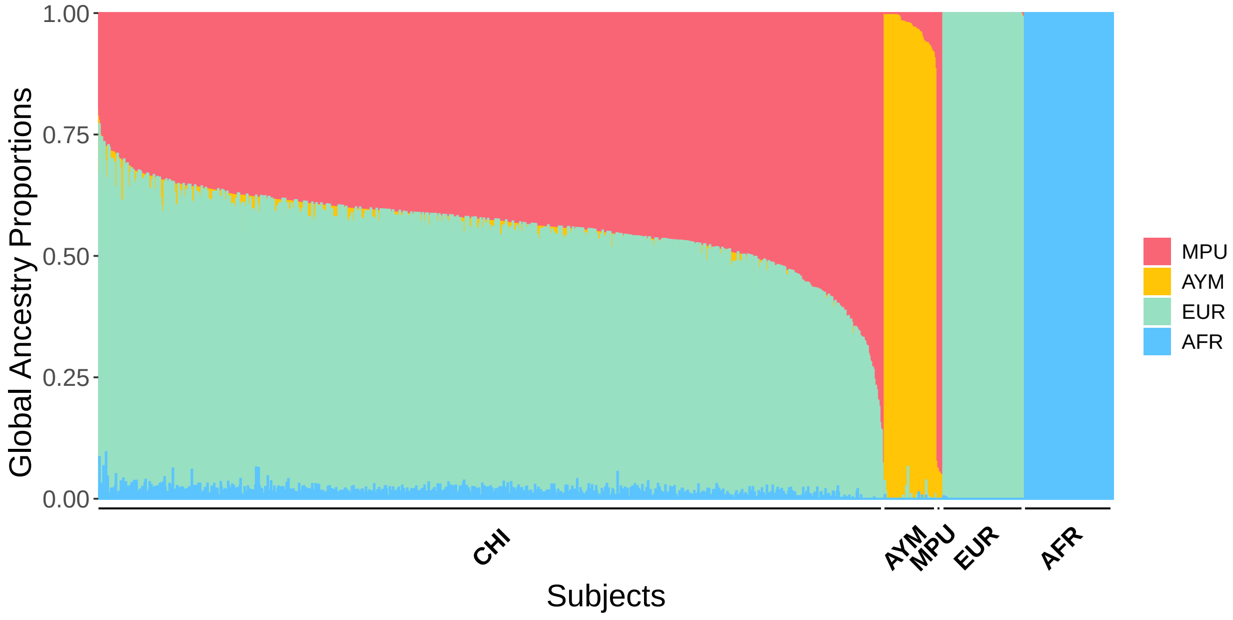

GWAS of PV, APV and HAPV. We performed GWAS for PV, APV and HAPV. In the regression model, we adjusted for gender, Native American local ancestry, Mapuche global ancestry, genotype, an interaction effect between gender and genotype and an interaction effect between gender and Native American local ancestry. To increase the power to detect significant associations, all regressions were performed in boys and girls pooled together. Importantly, however, the interaction effects in our analyses allowed to separate the effects of the genotype and local ancestry on just boys, just girls or both (see Methods). In the analysis in boys, we found 2 variant alleles, *rs75297609-T* and *rs57205007-G*, achieving the genome-wide association significance threshold of $P < 5 \times 10^{-8}$ for PV (**Table 1**). These two variants are located close to the *RN7SKP143* gene (**Table 1**), a pseudogene of the *7SK* gene with unknown function. The 2 associated variants, as well

as *RN7SKP143*, are in turn located within the copy number variant (CNV) *esv3624175* (**Figure 3**), and thus likely represent the same association signal. Indeed, the genotypes of *rs75297609* and *rs57205007* among individuals show a Pearson's correlation coefficient of 0.78. *rs75297609-T* and *rs57205007-G* have 8 and 13 carriers, respectively, as well as allele frequencies of 0.004 and 0.007, respectively. **Table 1** also shows 6 variants achieving nominal significance (P <10^{-6}).

We did not find significant associations for APV. However, one variant, *rs148840332*, located in the *RP11-184E9.1* lincRNA gene, was associated with APV at P =3 x 10^{-7} (**Supplementary Table** 1). In the GWAS for HAPV we did not find associations achieving nominal significance (P <10^{-6}; data not shown). In the analysis in girls (see Methods), we did not find significant associations for any phenotype. The Manhattan plots for the PV and APV GWAS are shown in **Figure 4** and **Supplementary Figure** 1, respectively.

SNP ID-Allele	Conseq	Biotype	Gene	$ß_{GT}$	P_{GT}	$ß_{GTxSex}$	P_{GTxSex}	$ß_{GTxLA}$	P_{GTxLA}
rs75297609-T	upstream	miscRNA	*RN7SKP143* (98Kb)	-2.72	1.2E-08	3.17	2.3E-06	2.08	5.4E-03
rs57205007-G	upstream	miscRNA	*RN7SKP143* (86Kb)	-2.13	3.7E-08	2.27	2.2E-06	1.13	2.4E-03
rs113497890-G	intron	protein	*LCOR*	-3.16	6.3E-08	3.05	1.9E-05	-0.45	6.2E-01
rs12240935-C	upstream	miscRNA	*Y_RNA* (24Kb)	-3.09	1.2E-07	2.91	3.6E-03	2.36	3.9E-03
rs143615510-A	intron	protein	*CCDC77*	3.60	4.7E-07	-2.66	1.2E-03	-1.86	2.3E-02
rs79659566-A	upstream	protein	*DYRK2* (37Kb)	-0.92	5.5E-07	0.41	2.4E-02	0.51	2.0E-05
rs6699943-C	intron	protein	*GLUL*	1.89	6.6E-07	-1.15	1.4E-02	-2.35	3.0E-07
rs112103074-A	intergenic	-	*AC011366.3* (-197Kb)	-1.21	8.3E-07	1.47	3.6E-06	0.76	1.7E-02

Table 1. Strongest associations for PV in boys. Shown are the SNP rs ID with the associated allele, the Sequence Ontology consequence type, biotype, closest gene (with the distance from the gene in Kb), effect size of the genotype (β_{GT}), association *P*-value of the genotype (P_{GT}), effect

size of the interaction between genotype and sex (β_{GTxSex}) with the corresponding association P-value (P_{GTxSex}), effect size of the interaction between genotype and Native American local ancestry (β_{GTxLA}) with the corresponding association P-value (P_{GTxLA}). The coding for sex was 1 for females and 0 for males. Therefore, the effect of the SNP allele in males corresponds to the β_{GT} column, and the effect of the SNP allele in females is the sum of the effects in the β_{GT} and β_{GTxSex} columns. Note that these effects are opposite in sign, which leads to a non-significant effect for females. Local ancestry is coded as 0, 1 or 2 depending on the number of alleles at each SNP that originate from Native America.

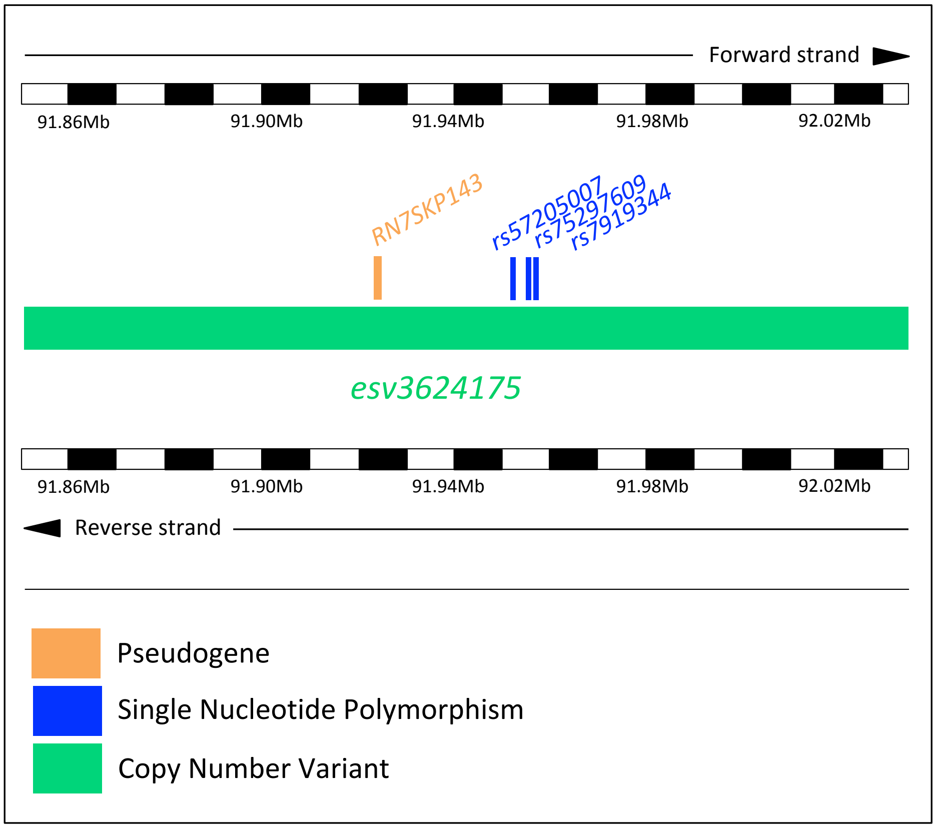

[H]

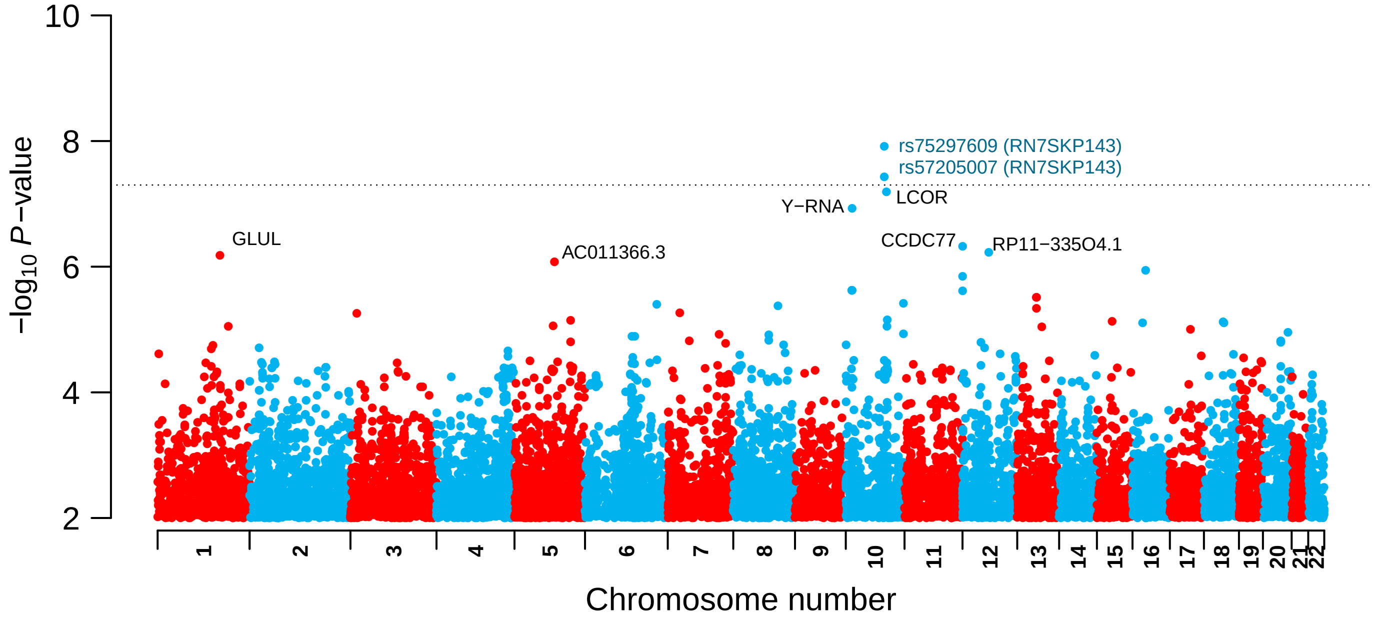

To interrogate more variants for putative associations with PV, we performed genotype imputations of SNPs located within ± 500 Kb **of the association signal harboring *rs75297609* and *rs57205007***. We used the Trans-Omics for Precision Medicine (TOPMed) server [20], which has a panel of $97,256$ samples from diverse ethnic backgrounds, including South Americans. We performed linear regressions adjusting for gender, Mapuche global ancestry, genotype and an interaction effect between gender and genotype. We did not include Native American local ancestry as covariate, since we lacked this information for imputed SNPs. In the analysis in boys, we found that the intergenic variant allele *rs7919344-T* was significantly associated with PV ($P = 1 \times 10^{-9}$; **Figure 5** and **Table** 2). We also found 7 intergenic SNPs achieving nominal genome-wide sig-

nificance ($P < 10^{-6}$; **Table** 2), including the previously associated *rs75297609-T* variant allele. All of these variants seem to be part of the same peak (**Figure 5**). Indeed, *rs7919344* is located 399 bp from *rs75297609* and 11.8 Kb from *rs57205007*. *rs7919344-T* has 7 carriers and an allele frequency of 0.004. In the analysis in girls, we did not find significant associations between PV and the imputed SNPs from chromosome 10.

SNP ID-Allele	Location	Gene	ß$_{GT}$	*P*$_{GT}$	ß$_{GTxSex}$	*P*$_{GTxSex}$
rs7919344-T	92021973	*RN7SKP143* (98Kb)	-2.38	8.0E-09	2.86	4.9E-06
rs148396815-C	92012923	*RN7SKP143* (89Kb)	-1.87	4.2E-07	2.60	6.1E-07
10:92014488-T	92014488	*RN7SKP143* (91Kb)	-1.87	4.4E-07	2.35	9.2E-05
rs184996157-A	92018837	*RN7SKP143* (95Kb)	-1.87	4.4E-07	2.35	9.2E-05
rs534950014-G	92011098	*RN7SKP143* (87Kb)	-1.87	4.4E-07	2.35	9.2E-05
rs560385300-T	92013183	*RN7SKP143* (89Kb)	-1.87	4.4E-07	2.35	9.2E-05
rs75297609-T	92021574	*RN7SKP143* (98Kb)	-1.87	4.4E-07	2.35	9.2E-05
10:92016131-A	92016131	*RN7SKP143* (92Kb)	-1.87	4.4E-07	2.08	1.7E-04

Table 2. Stronger associations for PV in boys using imputed genotypes surrounding the association signal. Shown is the SNP rs ID with the associated allele, physical location, gene, effect size of the genotype (β_{GT}), association *P*-value of the genotype (P_{GT}), effect size of the interaction between genotype and sex ($\beta_{GT \times Sex}$) with the corresponding association *P*-value ($P_{GT \times Sex}$). The effect of the SNP allele in males corresponds to the β_{GT} column, and the effect of the SNP allele in females is the sum of the effects in the β_{GT} and $\beta_{GT \times Sex}$ columns. Since these effects are opposite in sign, this leads to a non-significant effect for females. The region analyzed has physical coordinates 10:91521574-92510199.

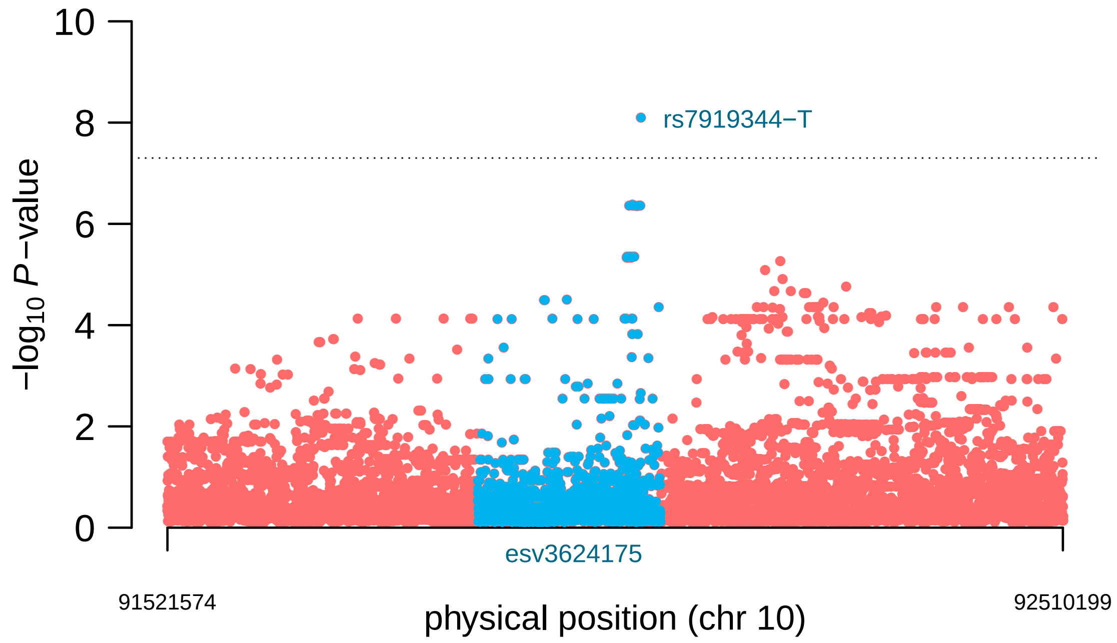

Effect of genetic ancestry on pubertal growth phenotypes. We analyzed whether PV, APV and/or HAPV are influenced by global genetic ancestry proportions. We found that Mapuche Native American global ancestry had average effect sizes of –0.73 years (P = 0.02) for APV and –4,3 cm (P = 0.04) for HAPV in boys and girls combined (**Figure 6**). Global ancestry did not have a significant effect on PV.

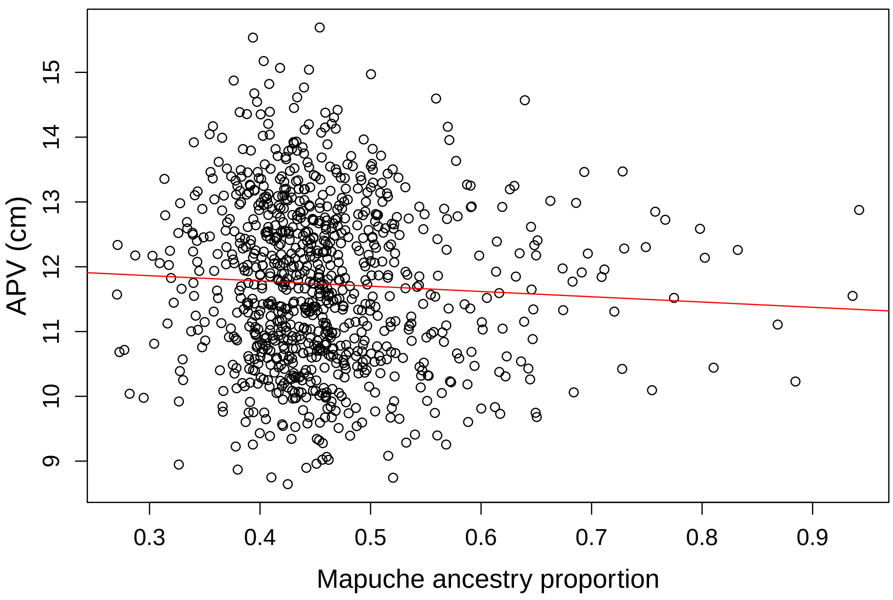

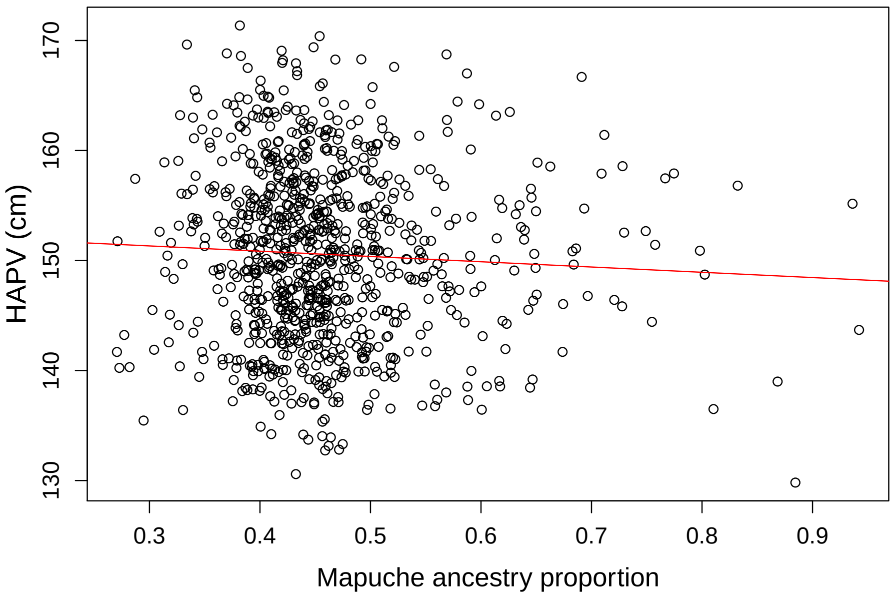

We also evaluated the effect of per-SNP wise local ancestry over PV and APV in non-imputed genotypes (HAPV was not analyzed due to an absence of strong GWAS associations). In the

regression model we measured the effect of Native American local ancestry and also the interaction term between genotype and Native American local ancestry (see Methods). The variant allele with the strongest genome-wide association with PV for the interaction term between genotype and Native American local ancestry was *rs6699943-C* (P = 3 x 10^{-7}; **Table 1**). We also found strong associations for the two significant variants in the GWAS for PV (P = 0.002 for *rs57205007-G* and P = 0.005 for *rs75297609-T*; **Table 1**).

Discussion

In this study, we aimed to better understand the genetic architecture of pubertal growth. We conducted GWAS on PV, APV and HAPV. The two former traits are critical antropometric markers of pubertal growth [21] [22]. However, besides a few associations studies between candidate genetic variants and PV in Europeans [22], we are not aware of previous GWAS on PV, APV or HAPV.

Our GWAS on **called and imputed SNPs detected a single genetic signal overlapping the copy number variant (CNV) *esv3624175*, which harbors the significantly associated variants *rs75297609-T*, *rs57205007-G* and *rs7919344-T***. This suggests that a causal variant linked to this region is driving the association with PV. This hypothesis is also supported by the high correlation among the genotypes of *rs75297609* and *rs57205007*. The associated variants are located close to the *RN7SKP143* pseudogene, which has unknown function. Even though the biological functions of the >200 *7SK* pseudogenes are unknown, some of them might have growth-related functions. For instance, on the same chromosome 10, *RN7SKP167* has been GWAS-associated with growth-

related phenotypes, including bone mineral density and hip bone size ($P = 4 \times 10^{-11}$) [23]. However, most pseudogenes are not functional, so it is also possible that the three significant GWAS associations could result from these variants being in high linkage disequilibrium with causal variants located in or around other growth-related genes. Unfortunately, there is no recombination map for the Chilean population to test this hypothesis.

We found an intron variant allele of the *LCOR* gene, namely, *rs113497890-G* –also on chromosome 10– reaching a *P*-value very close to genome-wide significance (P = 6.3 x 10^{-8}). This gene has been GWAS-associated with adult height (P = 4 x 10^{-20}) and heel bone mineral density (P = 1x 10^{-8}) [24], two phenotypes likely related with pubertal height growth. Increasing our sample size may augment statistical power to detect a significant association of the *LCOR* variant. It remains to be elucidated whether the main effects of the variants associated with PV on body growth would result from their neuro-endocrine actions through the Hypothalamic-Pituitary-Gonadal (HPG) axis that triggers puberty [25], or rather over local growth factors responsible for tissue growth. It is also unclear what biological mechanism underlies the sex-specific effects of the associated variants.

We analyzed the relationship between longitudinal pubertal traits and genetic ancestry. There are very few studies addressing this topic. An epidemiological study performed in a multiethnic US cohort showed that the mean APV is 13.7 years in boys and 12.1 years in females [21]. Our results show that Chilean children have a lower mean APV (12.7 years in boys and 10.8 years in girls). However, since that study on US children did not consider genetic variables, it is not possible to

estimate whether or not ancestry contributes to these differences. In our study of Chilean children, whose range for Mapuche ancestry varied from 0.25 to 0.94, we obtained that a difference in ancestry proportion led on average to a 0.73 years decrease in APV between a European and a Mapuche Native American boy. To our knowledge, we are the first to quantify how Native American genetic ancestry affects pubertal growth phenotypes, specifically PV, APV and HAPV. It is also important to recognize that differences in height growth and puberty milestones by ethnicity are not only determined by genetic ancestry, but also are influenced by social disparities among ethnic groups [6].

Mapuche and Aymara diverged from their common ancestral population ∼8750 years ago [26]. Thus, it is possible that long-time exposure to different environments as well as demographic forces led to changes in their genetic diversity, which in turn could have differentially affected pubertal growth-related phenotypes in a population-specific manner. However, to our knowledge, comparisons in pubertal growth between Mapuche and Aymara children have never been formally tested. By accounting for Mapuche and Aymara global ancestries in the GWAS linear regressions instead of considering them as a single ancestral component, we obtained more reliable GWAS associations, since most of the Native American ancestry of the Chilean children is Mapuche. Noteworthy, we found that the strongest genome-wide association for the interaction between genotype and local ancestry is *rs6699943-C*, an intron variant allele of the *GLUL* gene, which has not been GWAS associated with height growth-related traits.

Unfortunately, we did not replicate our findings in an independent cohort, since there is no

other longitudinal paediatric growth cohort with admixed Native American and European ancestries. European cohorts such as those that comprise the Early Growth Genetics Consortium [27], are not useful for replication, since the three associated variant alleles are only found in Africans and in **Latino populations (when considering populations from 1000G)** [28]. An exception is IBS (**Spaniards**) from 1000G, where *rs75297609-T* and *rs57205007-G* are found with allele frequencies of 0.005 and 0.009, respectively (information retrieved from the Variant Effect Predictor [29]). None of these variants are present in our Mapuche or Aymara reference panels. **Future research will be needed to determine the ancestral origin of the genetic signal harboring the three associated variants**.

The results of our study highlight the importance of including genetic ancestry when performing GWAS in populations of admixed ancestries, such as Latinos or African Americans. Finally, since pubertal growth traits significantly vary with genetic ancestry, GWAS on understudied longitudinal growth traits hold great potential to identifying loci playing functional roles in particular continental populations.

Subjects and Methods

Sample. We analized individuals of the "Growth and Obesity Chilean Cohort Study" (GOCS) [30]. This is a longitudinal follow-up of 1195 individuals recruited in 2006 (aged 3.5 years old) at different childcare centers from the South East Area of Santiago, Chile. The population represents a middle-low socioeconomic level and all participants were singleton births occurring during

$2002-2003$, who had birth weights between 2500 and 4500 g, with no medical or mental conditions. Annual measurements were carried out at Instituto de Nutrición y Tecnología en Alimentos (INTA), Santiago, Chile, by trained dietitians, and since 2010 visits took place every 6 months. We used standardized protocols and height was measured with a portable stadiometer (Harpenden 603; Holtain LTD, Crosswell, UK) to the nearest 0.1 cm.

Genotyping. We used genome-wide data of 848 individuals from Tobar *et al.*, 2019 [31] and genotyped 105 additional individuals for the present study. Genotype data was obtained using the Infinium ® Multhi-Ethnic Global BeadChip (Illumina). Genotyping was performed in the Human Genotyping laboratory at the Spanish National Cancer Research Centre, a member of CeGen (PRB2-ISCIII). Raw intensity genotype files were loaded into GenomeStudio v2.0.3 (Illumina) and automatic clustering was performed. We removed 18 samples with call rate < 0.98 from the total of samples genotyped. We also excluded variants with heterozygous genotypes on the X chromosome for males and variants calling genotypes on Y chromosome for females. Using PLINK 1.9 [32] we removed 10 additional samples based on gender mismatch, heterozygosity rate (variants with $\pm$3 SD from the mean heterozigosity) and ancestry outliers. For relatedness, we randomly discarded 1 person from each pair with IBD/IBS$>$0.2, corresponding to a kinship relationship between second and third degree. Excluded variants had missing genotype data $> 5\%$, duplicated physical positions (one variant was kept from each duplicate pair) and deviations from Hardy-Weinberg Equilibrium (HWE) ($P < 1$ x 10^{-6}). A-T and C-G tranversions were also excluded to avoid inconsistencies with the reference human genome (build GRCh37). We also excluded 25 boys whose last measurements were taken before they were 12 years old. After applying these

filters, we obtained a clean data set of 904 individuals and $774,433$ autosomal variants.

Local Ancestry Estimation. We used RFMix [33] to infer the local ancestry of genomic fragments in the 904 Chilean individuals. As reference populations for the local ancestry analysis we used the following populations from the 1000 Genomes Project [28]: Yoruba (YRI, n = 108) for African ancestry, Utah Residents with Northern and Western European Ancestry (CEU, n = 99) for European ancestry, and Peruvian (PEL, n = 85) for Native American ancestry. We merged all sets obtaining 1196 individuals and the $774,433$ SNPs. We inferred the gametic phase of individuals using Beagle 5 software [34], and the HapMap37 human genome build 37 recombination map. We used `PopPhased -n 5` and `--forward-backward` parameters as recommended in the RFMix manual.

Global Ancestry Estimation. We estimated the global ancestry proportions with ADMIXTURE [19], using K = 4 ancestral populations. As reference Native American populations, we included 7 Mapuche individuals from Vidal *et al.*, 2019 [35] and 64 Aymara individuals from Lindo *et al.*, 2018 [26] and Crawford *et al.*, 2017 [36]. As proxies for the European and African components, we used 99 individuals with Northern/Western European ancestry (CEU) and 102 Yoruba individuals from the 1000G Project [28], respectively.

Genotype Imputations. SNPs were imputed using the TOPMed Server [20], which includes a panel of 97,256 deeply sequenced human genomes. Genotypes aligned to the GRCh37/h19 assembly were submitted into the server and imputations were run with default parameters. Imputed genotypes were converted from GRCh38 to GRCh37/h19 assembly using CrossMap [37]. Chain files as

well as the reference genome were obtained from the UCSC Genome Browser [38]. Imputed genotypes were filtered by a R-square value of 0.5. Indels were removed and assignment of Reference ID (RS number) were based on 1000G's SNPs.

Longitudinal model. We estimated the growth curves for each individual in the sample through non-linear longitudinal mixed models; one model fitted to the girls and another model fitted to the boys, as implemented in the R package SITAR [39]. The model takes the form $y_{it} = \alpha_i + h(\frac{t-\beta_i}{exp(\gamma_i)})$, where α_i, β_i and γ_i are random effects for individual i, h is a natural cubic spline function of age (t in the model) versus height, where y_{it} represents the height of individual i ($i = 1, \ldots, n$) at time t. The fitted curves for each individual were used to calculate peak height velocity (PV), age at peak height velocity (APV) and height at the age of peak velocity (HAPV) using the function `getapv` [39].

GWAS for PV, APV and HAPV. For each of the three phenotypes we performed genome-wide associations by implementing linear regression models. The model included boys and girls together. We adjusted for gender, Native American local ancestry at each SNP, global ancestry for each individual and the genotype for each SNP. Global ancestry refers to the proportion of global Mapuche Native American ancestry and local ancestry was measured for each child at each SNP, and it consists of an estimate of the number of alleles that originate from Native America. It takes values $0, 1$ or 2. We also included in the model an interaction effect between genotype and local ancestry and an interaction effect between genotype and gender. The linear regression equation for the model was the following:

$$ph_i = \beta_0 + \beta_1 X_{1i} + \beta_2 X_{2i} + \beta_{3j} X_{3ij} + \beta_{4j} X_{4ij} + \beta_{5j} X_{1i} \times X_{4ij} + \beta_{6j} X_{1i} \times X_{3ij} + \epsilon_i$$

where X_1 is a dichotomous variable that represents male gender with 0 and female gender with 1. X_2 represents the proportion of global Mapuche Native American ancestry (this variable takes values between 0 and 1), X_{3j} represents the local Native American ancestry at SNP j (takes values 0, 1 or 2 that represent the number of alleles that originate from Native Americans), and X_{4j} corresponds to the additive genotype representation of SNP j. ph represents the phenotype that can take on the three phenotypes that we consider (PV, APV and HAPV) and the subscript i stands for measurements at individual i. Note that the genetic association of SNP j on female children is given by $\beta_{4j} + \beta_{5j}$, while the association on male children is β_{4j}. To identify significant genetic variants we performed three test of hypotheses for each phenotype and each SNP j: i) $H_0 : \beta_{4j} + \beta_{5j} = 0$ vs $H_1 : \beta_{4j} + \beta_{5j} \neq 0$; ii) $H_0 : \beta_{4j} = 0$ vs $H_1 : \beta_{4j} \neq 0$; iii) $H_0 : \beta_{5j} = 0$ vs $H_1 : \beta_{5j} \neq 0$.

To test associations between imputed SNPs and PV, we implemented a regression model similar to the aforementioned model, but we excluded the effect of Native American local ancestry. The linear regression equation for the model was the following:

$$ph_i = \beta_0 + \beta_1 X_{1i} + \beta_2 X_{2i} + \beta_{4j} X_{4ij} + \beta_{5j} X_{1i} \times X_{4ij} + \epsilon_i$$

The symbols of the covariates, their effects and the hypotheses tested were described in the previous model.

Variant and gene annotations. Variant annotations corresponding to the GRCh37 (hg19) assembly were retrieved with the web tool Variant Effect Predictor (VEP) from Ensembl [29]. They included the Sequence Ontology (SO) consequence type and Gencode biotypes. Upstream and downstream variants were defined as those located 10 Kb upstream or downstream of the gene, respectively. Intergenic variants were defined as those located >100 Kb upstream or downstream of the closest gene. We used HaploReg v4.1 [40] to identify the genes located closest to associated intergenic variants. Reported GWAS associations were retrieved from the NHGRI GWAS Catalog [41]. Only variants achieving the genome-wide associations threshold of $P < 5 \times 10^{-8}$ were considered. When more than one variant in a gene has been associated with the same phenotype, we reported the strongest association.

Competing interests

The authors declare that they have no competing financial interests.

Funding

This work was supported by the Fondo Nacional de Ciencia y Tecnología (FONDECYT) [3170038 and 11200324 to L.V.; 1190346 to V.M.; 1160833 and 1200146 to S.E., T.N. and JP.M; 1150416 and 1150486 to JL.S.]. S.E., T.N. and L.V. were additionally supported by the Instituto Milenio de Investigación Sobre los Fundamentos de los Datos (IMFD). The genotyping, led by JL.S., was supported by grant PT13/0001/0005 of PE I + D + i 2013-2016, funded by ISCIII and ERDF (Fondo Europeo de Desarrollo Regional).

Author's contributions

S.E. conceived the project. S.E., T.N., L.V., JP.M, L.O. and F.M. analyzed the data. A.P. and V.M. collected data. JL.S. and S.E. raised funds for genotyping, JL.S. supervised the collection of DNA samples for genotyping. L.V. wrote the manuscript, with contributions from S.E. All authors critically reviewed and accepted the final version.

Availability of data and material

We can provide summary data upon request.

Consent to Participate

Consent to participate and publish was obtained from parents or guardians and children assented to participate when they turned 7 years of age. The study was approved by the Scientific Ethics Committees of Instituto de Nutrición y Tecnología en Alimentos (INTA) and Pontificia Universidad Católica de Chile.

References

1. Day, F. R. *et al.* Shared genetic aetiology of puberty timing between sexes and with health-related outcomes. *Nat Commun* **6**, 8842 (2015).

2. Biro, F. M. *et al.* Pubertal correlates in black and white girls. *J Pediatr* **148**, 234–40 (2006).

3. Cousminer, D. L. *et al.* Genome-wide association and longitudinal analyses reveal genetic loci linking pubertal height growth, pubertal timing and childhood adiposity. *Hum Mol Genet* **22**, 2735–47 (2013).

4. Day, F. R., Elks, C. E., Murray, A., Ong, K. K. & Perry, J. R. B. Puberty timing associated with diabetes, cardiovascular disease and also diverse health outcomes in men and women: the uk biobank study. *Sci Rep* **5**, 11208 (2015).

5. Euling, S. Y. *et al.* Examination of us puberty-timing data from 1940 to 1994 for secular trends: panel findings. *Pediatrics* **121 Suppl 3**, S172–91 (2008).

6. Idossa, D., Duma, N., Chekhovskiy, K., Go, R. & Ailawadhi, S. Commentary: Race and ethnicity in biomedical research - classifications, challenges, and future directions. *Ethn Dis* **28**, 561–564 (2018).

7. Soliman, A., De Sanctis, V., Elalaily, R. & Bedair, S. Advances in pubertal growth and factors influencing it: Can we increase pubertal growth? *Indian J Endocrinol Metab* **18**, S53–62 (2014).

8. Herbison, A. E. Genetics of puberty. *Horm Res* **68 Suppl 5**, 75–9 (2007).

9. Parent, A.-S. *et al.* The timing of normal puberty and the age limits of sexual precocity: variations around the world, secular trends, and changes after migration. *Endocr Rev* **24**, 668–93 (2003).

10. Chan, Y. *et al.* Genome-wide analysis of body proportion classifies height-associated variants by mechanism of action and implicates genes important for skeletal development. *Am J Hum Genet* **96**, 695–708 (2015).

11. Styrkarsdottir, U. *et al.* Gwas of bone size yields twelve loci that also affect height, bmd, osteoarthritis or fractures. *Nat Commun* **10**, 2054 (2019).

12. Paternoster, L. *et al.* Adult height variants affect birth length and growth rate in children. *Hum Mol Genet* **20**, 4069–75 (2011).

13. Mills, M. C. & Rahal, C. The gwas diversity monitor tracks diversity by disease in real time. *Nature Genetics* **52**, 242–243 (2020). URL `https://doi.org/10.1038/s41588-020-0580-y`.

14. Elks, C. E. *et al.* Thirty new loci for age at menarche identified by a meta-analysis of genome-wide association studies. *Nat Genet* **42**, 1077–85 (2010).

15. Cousminer, D. L., Widén, E. & Palmert, M. R. The genetics of pubertal timing in the general population: recent advances and evidence for sex-specificity. *Curr Opin Endocrinol Diabetes Obes* **23**, 57–65 (2016).

16. Eyheramendy, S., Martinez, F. I., Manevy, F., Vial, C. & Repetto, G. M. Genetic structure characterization of chileans reflects historical immigration patterns. *Nat Commun* **6**, 6472 (2015).

17. Vicuña, L. *et al.* Post-admixture selection on chileans targets haplotype involved in pigmentation and immune defense against pathogens. *Genome Biol Evol* (2020).

18. Lorenzo Bermejo, J. *et al.* Subtypes of native american ancestry and leading causes of death: Mapuche ancestry-specific associations with gallbladder cancer risk in chile. *PLoS Genet* **13**, e1006756 (2017).

19. Alexander, D. H. & Lange, K. Enhancements to the admixture algorithm for individual ancestry estimation. *BMC Bioinformatics* **12**, 246 (2011).

20. Taliun, D. *et al.* Sequencing of 53,831 diverse genomes from the nhlbi topmed program. *Nature* **590**, 290–299 (2021).

21. Granados, A., Gebremariam, A. & Lee, J. M. Relationship between timing of peak height velocity and pubertal staging in boys and girls. *J Clin Res Pediatr Endocrinol* **7**, 235–7 (2015).

22. Sovio, U. *et al.* Genetic determinants of height growth assessed longitudinally from infancy to adulthood in the northern finland birth cohort 1966. *PLoS Genet* **5**, e1000409 (2009).

23. Zhang, H. *et al.* Pleiotropic loci underlying bone mineral density and bone size identified by a bivariate genome-wide association analysis. *Osteoporos Int* **31**, 1691–1701 (2020).

24. Kichaev, G. *et al.* Leveraging polygenic functional enrichment to improve gwas power. *Am J Hum Genet* **104**, 65–75 (2019).

25. Bordini, B. & Rosenfield, R. L. Normal pubertal development: Part i: The endocrine basis of puberty. *Pediatr Rev* **32**, 223–9 (2011).

26. Lindo, J. *et al.* The genetic prehistory of the andean highlands 7000 years bp though european contact. *Sci Adv* **4**, eaau4921 (2018).

27. Middeldorp, C. M. *et al.* The early growth genetics (egg) and early genetics and lifecourse epidemiology (eagle) consortia: design, results and future prospects. *Eur J Epidemiol* **34**, 279–300 (2019).

28. 1000 Genomes Project Consortium *et al.* A global reference for human genetic variation. *Nature* **526**, 68–74 (2015).

29. McLaren, W. *et al.* The ensembl variant effect predictor. *Genome Biol* **17**, 122 (2016).

30. Corvalán, C., Uauy, R. & Mericq, V. Obesity is positively associated with dehydroepiandrosterone sulfate concentrations at 7 y in chilean children of normal birth weight. *Am J Clin Nutr* **97**, 318–25 (2013).

31. Tobar, H. E. *et al.* Identification and functional analysis of missense mutations in the lecithin cholesterol acyltransferase gene in a chilean patient with hypoalphalipoproteinemia. *Lipids Health Dis* **18**, 132 (2019).

32. Purcell, S. *et al.* Plink: a tool set for whole-genome association and population-based linkage analyses. *Am J Hum Genet* **81**, 559–75 (2007).

33. Maples, B. K., Gravel, S., Kenny, E. E. & Bustamante, C. D. Rfmix: a discriminative modeling approach for rapid and robust local-ancestry inference. *Am J Hum Genet* **93**, 278–88 (2013).

34. Browning, B. L., Zhou, Y. & Browning, S. R. A one-penny imputed genome from next-generation reference panels. *Am J Hum Genet* **103**, 338–348 (2018).

35. Vidal, E. A. *et al.* Whole genome sequence, variant discovery and annotation in mapuche-huilliche native south americans. *Sci Rep* **9**, 2132 (2019).

36. Crawford, J. E. *et al.* Natural selection on genes related to cardiovascular health in high-altitude adapted andeans. *Am J Hum Genet* **101**, 752–767 (2017).

37. Zhao, H. *et al.* Crossmap: a versatile tool for coordinate conversion between genome assemblies. *Bioinformatics* **30**, 1006–7 (2014).

38. Kent, W. J. *et al.* The human genome browser at ucsc. *Genome Res* **12**, 996–1006 (2002).

39. Cao, Z., Hui, L. L. & Wong, M. Y. New approaches to obtaining individual peak height velocity and age at peak height velocity from the SITAR model. *Comput Methods Programs Biomed* **163**, 79–85 (2018).

40. Ward, L. D. & Kellis, M. Haploreg: a resource for exploring chromatin states, conservation, and regulatory motif alterations within sets of genetically linked variants. *Nucleic Acids Res* **40**, D930–4 (2012).

41. Welter, D. *et al.* The nhgri gwas catalog, a curated resource of snp-trait associations. *Nucleic Acids Res* **42**, D1001–6 (2014).

Figure 1 Estimation of PV and APV. The upper row shows the adjustment of the growth curves in boys (left) and girls (right), obtained using the SITAR package from R [39]. The lower row shows the mean height velocity (cm/year) in boys (left) and girls (right). The dotted vertical lines pinpoint the APV.

Figure 2 Global ancestry proportions. Each vertical line represents a subject and the colors the different ancestries. CHI: Chilean. The reference populations are MPU: Mapuche, AYM: Aymara, EUR: European; AFR: African.

Figure 3 *

Figure 4 Genomic context of the genetic signal associated with PV in boys. Shown is the copy number variant *esv3624175* harboring the significantly associated variants *rs75297609*, *rs57205007* and *rs7919344* as well as the pseudogene *RN7SKP143*. The image includes chromosome bands intercalated between black and white blocks with 10 Kb of size.

Figure 5 Manhattan plot for PV in boys. Genome-wide association *P*-values, expressed as -$\log_{10}$(*P*-value). Highlighted in blue are the 2 variants achieving genome-wide significant associations (with the associated gene names within parentheses) as well as the genes most closely located to variants with *P*-value $<10^{-6}$ (in black). Variants with -$\log_{10}$(*P*-value) between 0 and 2 are not shown.

Figure 6 *

Figure 7 Manhattan plot for PV in imputed SNPs from the region surrounding the copy number variant *esv3624175*. The region shown encompasses ± 500 Kb from *rs75297609* and *rs57205007*. Its physical coordinates are 10:91521574-92510199. Genome-wide association *P*-values are expressed as -$\log_{10}$(*P*-value). **Highlighted in blue is *rs7919344-T*, the significantly-associated variant allele located within *esv3624175*. All variants mapping *esv3624175* are labeled in blue. Variants with association *P*-value $<10^{-5}$ Table 2 also map *esv3624175* (not shown).**

Figure 8 Effect of global Mapuche ancestry on APV and HAPV. The top and lower panels show how APV and HAPV vary as a function of global Mapuche ancestry proportions in boys and girls considered together. The regression line is labeled in red.
